# Odor blocking of stress hormone responses

**DOI:** 10.1038/s41598-022-12663-x

**Published:** 2022-05-24

**Authors:** Eun Jeong Lee, Luis R. Saraiva, Naresh K. Hanchate, Xiaolan Ye, Gregory Asher, Jonathan Ho, Linda B. Buck

**Affiliations:** 1 Fred Hutchinson Cancer Research Center, 1100 Fairview Avenue North, A3-020, Seattle, WA 98109 USA; 2 Ajou University School of Medicine, Suwon, 16499 South Korea; 3 Present Address: Sidra Medicine, Research Branch, Out Patient Clinic, Doha, Qatar; 4 Present Address: University College London, London, UK; 5 Present Address: School of Medicine, University of Pittsburgh, S530 Alan Magee Scaife Hall, 3550 Terrace Street, Pittsburgh, PA 15261 USA

**Keywords:** Olfactory system, Stress and resilience, Neuroscience

## Abstract

Scents have been employed for millennia to allay stress, but whether or how they might do so is largely unknown. Fear and stress induce increases in blood stress hormones controlled by hypothalamic corticotropin releasing hormone neurons (CRHNs). Here, we report that two common odorants block mouse stress hormone responses to three potent stressors: physical restraint, predator odor, and male–male social confrontation. One odorant inhibits restraint and predator odor activation of excitatory neurons upstream of CRHNs in the bed nucleus of the stria terminalis (BNSTa). In addition, both activate inhibitory neurons upstream of CRHNs in the hypothalamic ventromedial nucleus (VMH) and silencing of VMH inhibitory neurons hinders odor blocking of stress. Together, these findings indicate that odor blocking can occur via two mechanisms: (1) Inhibition of excitatory neurons that transmit stress signals to CRHNs and (2) activation of inhibitory neurons that act directly or indirectly to inhibit stressor activation of CRHNs.

## Introduction

The mammalian olfactory system detects myriad volatile chemicals perceived as odors as well as animal cues that stimulate innate behavioral or physiological responses^1–3^. The historical use of odors^4^ to quell stress suggests that some odorants that alone elicit odor perceptions but not innate responses might nonetheless dampen innate responses to fear and stress. One hallmark of stress is a surge in blood stress hormones, which are controlled by corticotropin releasing hormone neurons (CRHNs) in the paraventricular nucleus of the hypothalamus^5,6^. A variety of stressors can induce stress hormone increases, including predator odors, physical restraint, injury, social stress^7,8^ and the activation of specific sensory neurons in the mouse olfactory epithelium^9^.

Previous studies suggested that CRHNs receive information from a number of brain areas^10^. In more recent studies, we used Cre-dependent Pseudorabies viruses to chart the locations of individual neurons upstream of CRHNs^11^. Neurons directly upstream of CRHNs were identified in 31 brain areas and were found to express a variety of neurotransmitters and neuromodulators^12,13^, including one linked to appetite suppression^13^. Neurons two synapses upstream of CRHNs were found in additional areas, including one olfactory cortical area that proved key to stress hormone responses to predator odors. Further studies revealed the locations of directly upstream neurons that are activated by two different stressors, physical restraint and a predator odor^13^.

In the present studies, we investigated the ability of common odorants to block stress hormone responses. We identified two odorants that block stress hormone responses to three diverse stressors: physical restraint, predator odor, and male-male social confrontation. Our studies show that odor blocking of stress can result from blocking odorant inhibition of neurons that transmit excitatory stressor signals to CRHNs in response to physical restraint or predator odor. They also reveal that blocking odorant activation of inhibitory neurons in one brain area may either directly or indirectly suppress CRHN responses to a stressor.

## Results

### Test odorants

**Figure 1:**
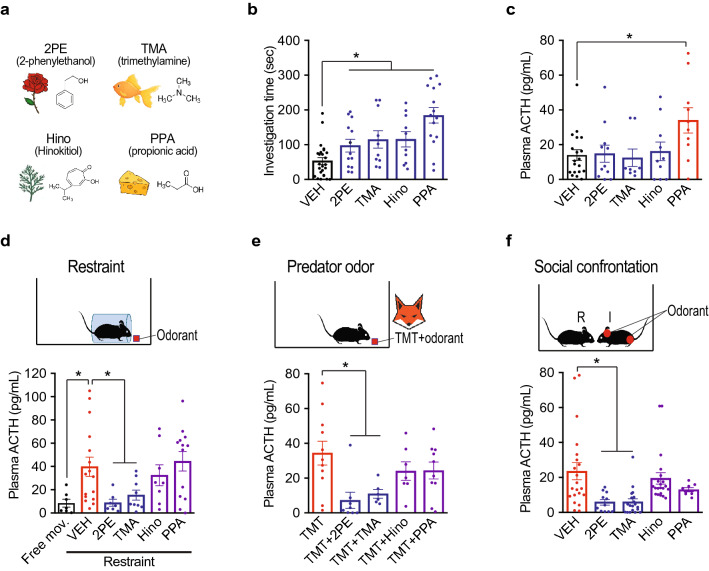
Certain odorants block stress hormone responses to diverse stressors. (**a–c**) Four odorants with different structures and perceived odors (**a**) were attractive (blue) to mice compared to vehicle (VEH) (gray) based on investigation time (**b**). Whereas one odorant (PPA, red) induced an increase in stress hormone (plasma ACTH) compared to vehicle, the others (blue) did not (**c**). n = 8–25 per condition. Unpaired t test, *P < 0.05. (**d**) Plasma ACTH was measured in freely moving animals (Free mov.) (gray) or in animals subjected to physical restraint in the presence of vehicle (VEH), 2PE, TMA, Hino, or PPA. Physical restraint caused an increase in stress hormone (red), which was blocked by exposure to 2PE or TMA (blue), but not Hino or PPA (purple). n = 7–16 per condition. Unpaired t test, *P < 0.05. (**e**) Predator odor (TMT, red) exposure stimulated an increase in plasma ACTH that was also blocked by 2PE or TMA (blue), but not Hino or PPA (purple). n = 6–19 per condition. Unpaired t test, *P < 0.05, **P < 0.01. (**f**) Social confrontation induced an increase in plasma ACTH when a resident male (R) was exposed to an intruder male (I) (VEH, red). Painting the intruder head and genital region with 2PE or TMA (blue), but not Hino or PPA (purple), blocked the stress hormone increase. n = 8–21 per condition. Unpaired t test, *P < 0.05. Column heights indicate means, error bars indicate S.E.M., and dots in the same column indicate different animals.

To test whether common odorants can block stress hormone responses, we sought odorants that are attractive to mice, but have dissimilar structures and perceived odor qualities. We selected four odorants: 2-phenylethanol (2PE) (rose)^14^, trimethylamine (TMA) (fishy)^15,16^, hinokitiol (Hino) (woody)^17^, and propionic acid (PPA) (cheese)^15^ (Fig. 1a). Filter paper containing each odorant increased investigation time compared to vehicle, confirming its attractiveness (Fig. 1b). With the exception of PPA, the odorants did not increase plasma levels of the stress hormone ACTH (adrenocorticotropic hormone) (Fig. 1c).

### Certain odorants block stress hormone responses to diverse stressors

Physical restraint is a potent stressor that induces large increases in stress hormone in mice^7^. To test for odorant effects on this response, mice were placed in a restrainer that restricts lateral and anterior–posterior movement but has a hole near the animal’s nose that allows it to smell an odorant placed on a nearby piece of filter paper. Mice were subjected to physical restraint in the presence of different odorants or vehicle for 10 min, and plasma ACTH was then measured. Two of the test odorants, 2PE and TMA, dramatically decreased restraint-induced ACTH, whereas the other two, Hino and PPA had no effect. Compared to vehicle, 2PE reduced plasma ACTH by 4.5-fold and TMA by 2.6-fold (Fig. 1d).

The fox predator odor TMT (2,5-dihydro-2,4,5-trimethylthiazoline) also induces stress hormone increases in mice^18^. To examine whether the test odorants affect this response, filter paper was placed at one end of the animal’s cage for 10 min that contained TMT, or TMT together with 2PE, TMA, Hino, or PPA. Strikingly, as with the ACTH response to restraint, both 2PE and TMA significantly inhibited the ACTH response to TMT, whereas neither Hino nor PPA did so. Plasma ACTH seen with TMT alone was decreased 4.8-fold by 2PE and 3.1-fold by TMA (Fig. 1e). These results are consistent with a report that rose oil inhibits the ACTH response to TMT^19^, but differ from a reported ability of a higher concentration of Hino to do so^17^.

Remarkably, 2PE and TMA also inhibited the stress hormone response to a third stressor, male-male social confrontation^8^. Male mice (“residents”) were singly housed and then exposed for ten minutes to an intruder male whose head and genital region were painted with an odorant or vehicle. Plasma ACTH in residents exposed to intruders painted with vehicle was reduced 4.1-fold when intruders were painted with 2PE and 3.9-fold when they were painted with TMA (Fig. 1f). In contrast, neither Hino nor PPA affected the stress hormone response to male-male social confrontation.

Together, these findings demonstrate that certain common odorants can block stress hormone responses. They further show that the same odorants can inhibit stress hormone responses to multiple different stressors. These effects are presumably due to the detection of odorants by olfactory sensory neurons in the nose, although a contribution by nasal trigeminal fibers, which can detect nasal irritants, cannot be excluded^20,21^.

### Blocking odorant inhibition of excitatory neurons upstream of CRHNs

How might an odorant block stress hormone responses? One possibility is that the blocking odorant inhibits stressor activation of neurons that transmit stressor signals to CRHNs. This would inhibit stressor activation of CRHNs and prevent their induction of stress hormone increases.

**Figure 2:**
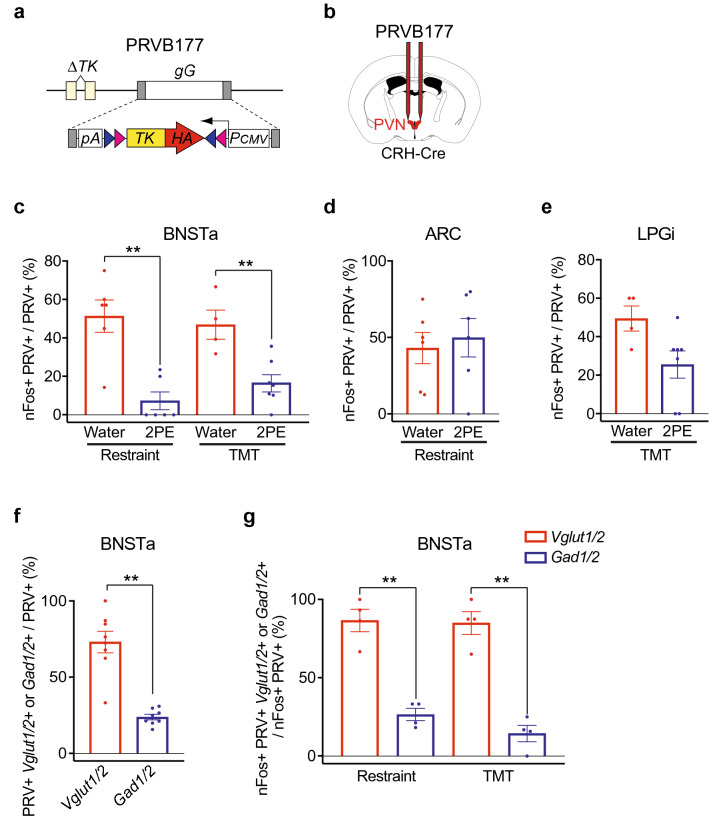
A blocking odorant inhibits stressor activation of BNSTa excitatory neurons upstream of CRHNs. (**a,b**) PRVB177, which has Cre-dependent expression of HA-TK (**a**), was used to infect Cre-expressing CRHNs in CRH-ires-Cre mice (**b**). (**c–e**) On d3pi of CRHNs with PRVB177, animals were exposed to restraint or TMT in the presence of 2PE or water. Brain sections were next costained for HA (PRV) and nFos mRNA. The percentage of PRV+ neurons in a given area that were nFos+ was then determined (nFos+ &PRV+/PRV+ (%)). 2PE significantly decreased the percentage of nFos labeled PRV+ neurons in BNSTa in response to restraint and TMT (**c**). However, 2PE did not significantly affect the percentage of nFos labeled PRV+ neurons in ARC with restraint (**d**) or those in LPGi with TMT (**e**). Column heights indicate means, error bars indicate S.E.M., and dots in the same column indicate different animals. n = 4–7 per condition. Unpaired t test, **P < 0.01. (**f,g**) A higher percentage of BNSTa PRV+ neurons were colabeled for Vglut1/2 than Gad1/2 (**f**). A large majority of BNSTa PRV+ neurons activated by restraint or TMT were colabeled for Vglut1/2 but not Gad1/2 (**g**). Column heights indicate means, error bars indicate S.E.M., and dots in the same column indicate different animals. n = 4 per condition. Unpaired t test, **P < 0.01.

To investigate this possibility, we focused on neurons directly upstream of CRHNs that are activated by either physical restraint or the fox predator odor TMT. Using CRH-ires-Cre mice^22^, we infected CRHNs with PRVB177 (Fig. 2a,b), a Pseudorabies virus that travels retrogradely across synapses and first infects directly upstream neurons on day 3 post-infection (d3pi)^11^. In previous studies, we exposed infected animals on d3pi to physical restraint or TMT and then costained brain sections for PRV and the neural activity marker nuclear Fos mRNA (nFos)^13^. Those studies showed that restraint activates neurons upstream of CRHNs in the BNSTa (bed nucleus of the stria terminalis) and ARC (arcuate nucleus of the hypothalamus) whereas TMT activates those in the BNSTa and LPGi (lateral paragigantocellular nucleus).

To examine whether a blocking odorant can inhibit stressor activation of neurons upstream of CRHNs in these areas, we infected CRHNs with PRVB177 and on d3pi exposed animals to restraint or TMT in the presence or absence of the blocking odorant 2PE. Brain sections were next costained for PRV and nFos. We then determined the number of PRV+ neurons and PRV+ nFos+ neurons in individual brain areas and calculated the percentage of PRV+ neurons that were nFos + in a given area (nFos+ &PRV+/PRV+ (%)).

2PE caused a striking decrease in the activation of upstream BNSTa neurons by both restraint and TMT (Fig. 2c). Whereas 51.4 ± 8.4% of PRV+ BNSTa neurons were nFos+ following restraint alone, 7.3 ± 4.6% were nFos+ following exposure to restraint plus 2PE, a decrease of sevenfold. And while 47.0 ± 7.6% of PRV+ BNSTa neurons were nFos+ after exposure to TMT, 16.7 ± 4.5% were nFos+ after exposure to TMT plus 2PE, a decrease of 2.8-fold.

In contrast, 2PE had no discernible effect on restraint activation of PRV+ neurons in the ARC and little or no effect on TMT activation of PRV+ neurons in LPGi (Fig. 2d,e). It has been reported that rose oil decreases TMT-induced neuronal activation in a part of BNST^19^. However, the present results show an effect of a monomolecular odorant on a specific subset of BNST neurons: BNSTa neurons upstream of CRHNs. They also show an inhibitory effect not only on the response to TMT, but also on the response to another stressor, physical restraint.

To determine whether the stressor activated neurons in the BNSTa are excitatory or inhibitory, tissue sections were costained for PRV, nFos, and markers of excitatory glutamatergic neurons (Vglut1 and Vglut2) or inhibitory GABAergic neurons (Gad1 and Gad2). These experiments showed that the majority of BNSTa neurons upstream of CRHNs are glutamatergic. Whereas 73.1 ± 7.1% of the PRV+ neurons were colabeled for glutamatergic makers only 23.8 ± 1.8% were colabeled for GABAergic markers (Fig. 2f).

Of neurons activated by restraint, 86.6 ± 7.2% were glutamatergic and only 26.5 ± 4.0% were GABAergic. And of neurons activated by TMT, 85.0 ± 7.3% were glutamatergic and only 14.4 ± 5.2% were GABAergic (Fig. 2g, Supplementary Fig. 1). Thus, the vast majority of BNSTa upstream neurons activated by either TMT or physical restraint are glutamatergic excitatory neurons.

Given that 2PE causes a sevenfold decrease in upstream BNSTa neurons activated by restraint and a 2.8-fold decrease in those activated by TMT (Fig. 2c), these results indicate that most of the neurons inhibited by 2PE are glutamatergic neurons that transmit excitatory signals to CRHNs when animals are subjected to physical restraint or TMT exposure.

These findings clearly indicate that odor blocking of stress can occur via a mechanism in which a blocking odorant inhibits the activation of excitatory neurons upstream of CRHNs. This inhibition would prevent the upstream neurons from transmitting stressor signals to CRHNs and thereby prevent stressor activation of CRHNs and their induction of a stress hormone response.

### Blocking odorant activation of inhibitory neurons upstream of CRHNs

The above studies showed that odor blocking of stress can result from blocking odorant inhibition of excitatory neurons that transmit stressor signals to CRHNs. Could a blocking odorant also inhibit CRHNs, for example by stimulating inhibitory neurons upstream of CRHNs?

To explore this possibility, we asked whether 2PE or TMA can activate inhibitory neurons directly upstream of CRHNs. CRHNs were infected with PRVB177, and animals were exposed on d3pi to 2PE, TMA, or vehicle. Brain sections were then costained for PRV and nFos and the percentage of PRV+ neurons that were nFos+ was determined for each brain area (nFos+ &PRV+/PRV+ (%)).

**Figure 3:**
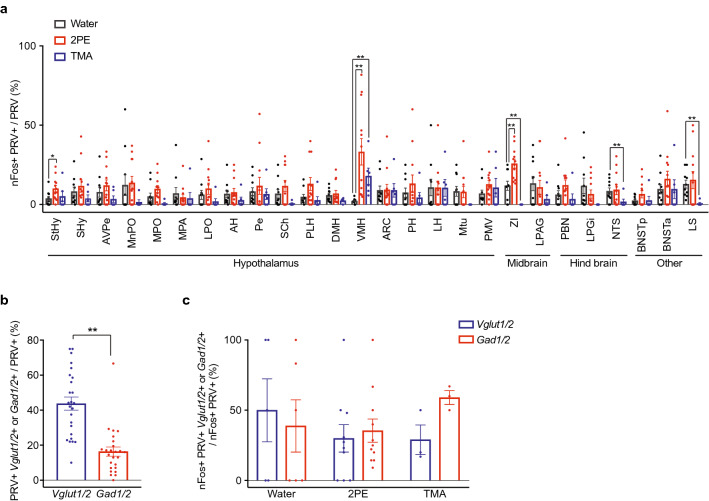
Blocking odorants activate inhibitory neurons upstream of CRHNs in VMH. (**a**) On d3pi after CRHN infection with PRVB177, animals were exposed to 2PE, TMA, or water and brain sections were costained for PRV and nFos. The percentage of PRV+ neurons in individual brain areas that were colabeled for nFos was then determined (nFos+ &PRV+/PRV+ (%). Column heights indicate means, error bars indicate S.E.M., and dots in the same column indicate different animals. n = 6–12 per condition. Unpaired t test, *P < 0.05, **P < 0.01. PRV+ neurons in the VMH showed significant activation by both 2PE and TMA. (**b,c**) Following exposure to water, 2PE, or TMA, VMH sections were costained for HA (PRV), nFos, and markers of glutamatergic neurons (Vglut1/2) or GABAergic neurons (Gad1/2). Many PRV+ VMH neurons were colabeled for either Vglut/2 or Gad1/2 (**b**). Both 2PE and TMA activated Vglut1/2 and Gad1/2+ PRV+ neurons in the VMH (**c**). Column heights indicate means, error bars indicate S.E.M., and dots in the same column indicate different animals. n = 3–12 per condition. Unpaired t test, **P < 0.01.

Significant activation of PRV+ neurons by both 2PE and TMA was seen in only one area, the ventromedial nucleus of the hypothalamus (VMH) (Fig. 3a). 2PE also caused significant activation of PRV+ cells in the StHy and ZI whereas TMA also caused significant decreases in activated cells in the ZI, NTS, and LS.

To examine whether the activated VMH neurons are excitatory or inhibitory, brain sections were costained for PRV, nFos, and Vglut1 and Vglut2 or Gad1 and Gad2. These experiments showed that VMH neurons upstream of CRHNs can be glutamatergic or GABAergic, although more are glutamatergic than GABAergic (Fig. 3b). They further showed that 2PE and TMA can activate both glutamatergic and GABAergic upstream neurons in the VMH (Fig. 3c, Supplementary Fig. 2).

These results suggested that both 2PE and TMA can activate VMH inhibitory neurons directly upstream of CRHNs to inhibit stressor activation of CRHNs, thereby contributing another mechanism to odor blocking. A potential role for blocking odorant activation of upstream excitatory neurons is unclear, although it could conceivably serve a homeostatic function by fine tuning the GABAergic inhibition of CRHNs.

### VMH inhibitory neurons can modulate stress responses

Could VMH GABAergic neurons contribute to odor blocking of stress? To examine this possibility, we asked whether chemogenetic activation or silencing of these neurons can alter the stress hormone response to physical restraint or its blocking by 2PE.

**Figure 4:**
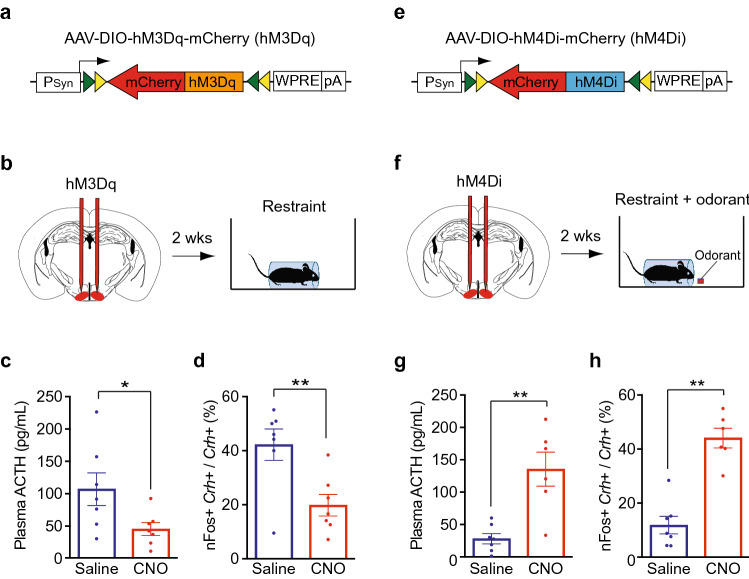
Silencing VMH GABAergic neurons affects odor blocking of stress. (**a,b,e,f**) AAVs with Cre-dependent expression of hM3Dq or hM4Di were used to activate (hM3Dq) (**a**) or silence (hM4Di) (**e**) VMH GABAergic neurons in Gad2-Cre mice. After ~ 2 weeks, animals were injected with CNO to activate hM3Dq or hM4Di, or with control saline, and then exposed to physical restraint (hM3Dq) (**b**) or physical restraint plus 2PE (hM4Di) (**f**). Plasma ACTH was then measured, and brain sections were costained for CRH and nFos. (**c,d**) Activation of VMH Gad2+ neurons with hM3Dq inhibits restraint-induced increases in ACTH and CRHN activation. Following restraint, CNO-treated animals showed significant decreases in plasma ACTH (**c**) and the percentage of Crh+ CRHNs stained for nFos (**d**) compared to animals treated with saline. Column heights indicate means, error bars indicate S.E.M., and dots in the same column indicate different animals. n = 7 per condition. Unpaired t test, *P < 0.05, **P < 0.01. (**g,h**) Silencing of VMH Gad2+ neurons with hM4Di interferes with the ability of 2PE to block restraint-induced increases in plasma ACTH and CRHN activation. In animals exposed to restraint plus 2PE, CNO treatment resulted in significant increases in plasma ACTH (**g**) and the percentage of CRHNs stained for c-Fos (**h**) compared to saline treatment. Column heights indicate means, error bars indicate S.E.M., and dots in the same column indicate different animals. n = 6–7 per condition. Unpaired t test, **P < 0.01.

Using Gad2-ires-Cre mice^23^, we infected Gad2+ neurons in the VMH with an adeno-associated virus (AAV) that has Cre-dependent expression of the hM3Dq or hM4Di receptor fused to mCherry (Fig. 4a,e). Upon binding to clozapine-n-oxide (CNO), hM3Dq activates neurons whereas hM4Di silences neurons^24^.

Following AAV infection, animals were injected with CNO or control saline and subjected to physical restraint (Fig. 4b) or physical restraint in the presence of the blocking odorant 2PE (Fig. 4f). Plasma ACTH was then measured, and brain sections were costained for CRH and Fos to assess CRHN activation. Brain sections were also immunostained for mCherry to determine the locations of neurons expressing hM3Dq or hM4Di.

Activation of GABAergic VMH neurons by hM3Dq significantly decreased restraint-induced ACTH. CNO-treated animals showed a 2.4-fold decrease in plasma ACTH relative to saline-treated controls (Fig. 4c). Consistent with this result, they also showed a 2.1-fold decrease in activated (nFos +) CRHNs, which are increased in response to restraint (Fig. 4d, Supplementary Fig. 3). Thus, activation of GABAergic VMH neurons inhibits restraint-induced increases in ACTH and CRHN activation.

Due to the difficulty of injecting AAV only into the small VMH, numerous animals also or instead showed mCherry + cells in nearby areas. Some animals with infected cells only in other areas also showed reduction in restraint-induced ACTH (Supplementary Fig. 4a). This is consistent with the presence of GABAergic neurons directly upstream of CRHNs in many brain areas^13^.

Silencing of VMH GABAergic neurons with hM4Di had a marked effect on the ability of 2PE to block restraint-induced ACTH and CRHN activation (Fig. 4f, Supplementary Fig. 3). Compared to controls, CNO-treated animals showed a 4.8-fold increase in ACTH and a 3.7-fold increase in activated CRHNs (Fig. 4g,h). ACTH increases were also seen in some animals with mCherry+ cells also or instead in other areas (Supplementary Fig. 4b). These results suggest that VMH GABAergic neurons can play a role in odorant blocking of the stress hormone response to restraint, but that GABAergic neurons in other areas may also be involved.

These findings are consistent with the idea that 2PE might block stress hormone responses in part by activating VMH GABAergic neurons upstream of CRHNs, and thereby suppressing CRHNs. However, the large-scale silencing of GABAergic VMH neurons in these studies undoubtedly goes far beyond the silencing of only those that are activated by 2PE. In addition, the effects of this silencing could be indirect. For example, it might block inhibitory signals transmitted from the VMH to neurons in other areas, such as the BNSTa, which send stressor signals to CRHNs. Further analyses will be needed to tease out the role of the VMH GABAergic neurons that are activated by 2PE in odor blocking of stress. Nonetheless, these studies suggest that VMH GABAergic neurons can play a direct or indirect role in the odorant blocking of stress.

## Discussion

In these studies, we asked whether common odorants can block stress responses in mice and, if so, how they might do so. We focused on stress-induced increases in blood stress hormones, which are controlled by hypothalamic CRHNs.

Our experiments demonstrate that certain common monomolecular odorants can indeed block stress hormone responses. Of four odorants attractive to mice, two blocked stress hormone responses. Moreover, the same two odorants inhibited stress hormone responses to three potent stressors: physical restraint, predator odor, and male-male social confrontation (Fig. 1). Because the two odorants have unrelated structures and perceived odor qualities (rose versus fishy to humans), it is unclear why they block stress hormone responses whereas the other two odorants do not. The most likely explanation is that odorants that block stress engage distinct neural circuits, but what those might be is unknown.

How does a single odorant block stress hormone responses to diverse stressors? Our studies indicate that odor blocking of stress can occur via the inhibition of excitatory neurons that send stressor signals to CRHNs. The predator odor TMT and physical restraint both activate excitatory neurons directly upstream of CRHNs in the BNSTa^13^. We found that the blocking odorant 2PE dramatically reduces the number of BNSTa neurons activated by both stressors. These results indicate that a blocking odorant can inhibit stress responses to both TMT and restraint by inhibiting stressor activation of BNSTa neurons upstream of CRHNs. As a consequence, the transmission of excitatory stress signals from those neurons to CRHNs is drastically reduced, thus preventing the activation of CRHNs and their induction of stress hormone responses to the stressors (Fig. 2).

Our experiments suggest that odor blocking of stress can also involve GABAergic inhibitory neurons in the VMH. We find that blocking odorants can activate VMH GABAergic neurons directly upstream of CRHNs (Fig. 3). Chemogenetic activation of VMH GABAergic neurons decreases the stress hormone response to restraint and their silencing interferes with the ability of 2PE to block this response (Fig. 4). These results suggest that VMH GABAergic neurons could play a role in odor blocking of stress either by directly inhibiting CRHNs or by inhibiting stressor activation of neurons upstream of CRHNs in other brain areas.

## Methods

### Mice

Mice aged 2–3 months were used. CRH-ires-Cre mice were generated previously^22^. C57BL/6J wildtype mice and GAD2-Cre mice (JAX 01082) were purchased from the Jackson Laboratory. All procedures involving mice were approved by the Fred Hutchinson Cancer Research Center Institutional Animal Care and Use Committee and carried out in accordance with ARRIVE guidelines. Mice were housed on a 12:12 h light:dark schedule (lights on at 07:00 AM) in a GM500 Tecniplast cages with an area of 501 cm^2^. Male mice were used in all experiments. No statistical methods were used to predetermine sample size. Animals were randomly chosen for experimental subjects. All the experiments except social confrontation were performed between 9:00 AM and 11:00 AM. Social confrontation was performed between 5:00 PM and 7:00 PM. The elapsed time between the first and last sacrifice for the experiments was always less than one hour. Animals were habituated to the institutional animal facility for at least 5 days after arrival. Adult male C57BL/6J mice used for restraint, odor exposure and social confrontation (except intruder male mice in Fig. 1) were maintained in single housed condition at least 10 days. Five intruder animals were group housed per cage. All animals used for PRV experiments shown in Figs. 2 and 3 were single housed 3 days after PRV injection (d3pi). The animals used for chemogenetic experiments shown in Fig. 4 were single housed until 2 weeks after AAV injection. Before single housing for PRV and chemogenetic experiments, up to 5 animals were kept in a single cage. Animals were excluded from certain experiments. For nFos experiments shown in Fig. 2, animals were excluded if PRV+ cells were not more than 10 cells in BNSTa. Three animals were excluded based on this criterion. For chemogenetic activation or silencing of the VMH, the animals used had mCherry+ cells indicative of viral infection only in the VMH.

### Viral vectors

#### PRV

PRVB177 was propagated following methods described previously^11,25^. Briefly, to propagate PRVB177, PK15 cells (ATCC) were infected with the virus using a multiplicity of infection (m.o.i.) = 0.1–0.01. After infection, cells showed a prominent cytopathic effect (~ 2 days). They were harvested by scraping, and the cell material was frozen using liquid nitrogen and then quickly thawed in a 37 °C water bath. After three freeze–thaw cycles, cell debris was removed by centrifugation twice at 1000×*g* for 5 min and the supernatant was then used for experiments. The titre of viral stocks was determined using standard plaque assays on PK15 cells, with titres expressed in plaque-forming units (p.f.u.).

#### AAVs

Serotype 8 AAVs were used with Cre recombinase-dependent flexstop cassettes that permit expression of mCherry-fused hM3Dq or mCherry-fused hM4Di under the control of the human synapsin promoter (AAV-DIO-hM3Dq-mCherry, AAV-DIO-hM4Di-mCherry)^26^. The viruses were purchased from the Vector Core at the University of North Carolina at Chapel Hill (the UNC vector core). Amount used is described in virus particles (v.p.).

### Stressors

#### Restraint

Mice were placed individually in a restrainer (a transparent plastic cylinder)^27^ located in their home cage for 10 min. The restrainer had a hole in the end near the tip of the animal’s nose. A piece of filter paper (1.5 × 2 cm) with absorbed 50 µL of odorant or VEH (water or DMSO) was placed in the cage near the hole in the cylinder.

#### Predator odor

Mice were exposed to a predator odor or distilled water as described previously^15^. A piece of filter paper (impregnated with 50 µL of 85 mM 2,5-dihydro-2,4,5-trimethylthiazoline (TMT) (Contech) diluted in water) was dropped gently into one end of the home cage. Animals were exposed to filter paper for 10 min.

#### Social confrontation

A male intruder (odorant-swabbed, see below) was placed in a resident’s home cage for 10 min.

#### Odor exposure

Olfactory investigation time tests (“olfactory preference test”) were conducted as described previously^15^, with minor modifications (see below). Adult male C57BL/6J mice were each assayed only once to avoid possible bias attributable to learning. On the day of testing^11^, mice were brought to the experimental room and habituated for 1 h. In the single odorant experiments, exposure to olfactory stimuli was done by gently dropping a piece of filter paper (1.5 × 2 cm) impregnated with 50 μL of vehicle (VEH, distilled water (H2O) for all odors except Hinokitiol or dimethyl sulfoxide (DMSO) for Hinokitiol) or odorant (85 mM in VEH, equivalent to 4.25 μmol), into one end of the cage. For the binary odorant mixture exposures, 25 μL of each odorant (4.25 μmol) was placed on a different^11^ end of the filter paper for a final concentration of 85 mM of each odorant. Behavioral tests were video recorded^11^ for 15 min^11^ with a Canon PowerShot ELPH300HS camera. The total olfactory investigation times were measured during 15 min of odorant exposure.

For ACTH assay in resident male mice confronted with odorant-swabbed intruders, resident male mice were brought to the experimental room and habituated for 1 h. Exposure to intruders with odorants was done by swabbing intruders with vehicle (VEH, distilled water (H2O) or dimethyl sulfoxide (DMSO)) or odorant (85 mM in VEH, equivalent to 4.25 μmol) on their heads (40 μL) and genitals (10 μL) prior to placing an intruder in the home cage of a single-housed male.

For analysis of c-Fos expression in CRH neurons, adult male C57BL/6J mice were exposed to water or 2PE with restraint for 10 min and then perfused 50 min later.

For detection of nFos in PRV-infected cells, CRH-Cre mice injected with PRVB177 3 days earlier were exposed to an odorant (85 mM) with/without stressors for 5 min.

#### Stereotaxic injection

Viruses were injected into the brain using a Stereotaxic Alignment System (David Kopf Instruments) with an inhalation anaesthesia of 2.5% isoflurane. Virus suspensions (PRVs: 1–1.5 × 10^6^ p.f.u. (1 μL); AAVs: 1–3 × 10^9^ v.p. (150 nL)) were loaded into a 1-μL syringe (Hamilton 80100 7001KH Syringe, 1 μL, 25 Gauge, 2.75″ Needle, Point Style 3), and injected at 100 nL per minute. The needle was inserted into the target location based on a stereotaxic atlas (AP: − 0.4, ML: ± 0.3, DV: − 5.0 mm for PVN; AP: − 1.5, ML: ± 0.78, DV: − 5.75 mm for VMH). After recovery, animals were singly housed with regular 12 h dark/light cycles, and food and water were provided ad libitum.

#### Plasma ACTH assay

Plasma ACTH assays were performed as described previously^11^. Briefly, after mice were killed by cervical dislocation and decapitation, trunk blood was collected directly into blood collection tubes (Becton Dickinson) containing 50 μL aprotinin (Phoenix Pharmaceuticals). Plasma was obtained by centrifugation at 1600×*g* for 15 min at 4 °C, and stored at − 80 °C. Plasma ACTH concentrations were measured using the ACTH ELISA kit (MD Biosciences), according to the manufacturer’s instructions, with the following modifications: (1) 100 μL of the controls or blood plasma combined with 100 μL of PBS (Phosphate Buffered Saline, pH 7.4) was used in the place of 200 μL plasma and (2) the results were assessed with the QuantaRed Enhanced Chemifluorescent HRP Substrate (Thermo Fisher). Fluorescence was measured with a CytoFluor4000 plate reader (Applied Biosystems). ACTH assays were carried out in duplicate. The average inter- and intra-assay coefficients of variation were 4.0% and 5.8%, respectively.

#### In situ hybridization and immunofluorescence

Conventional in situ hybridization was performed essentially as described previously, with some experiments using additional steps for triple staining. Coding region fragments of Vglut1, Vglut2, Gad1, Gad2, and c-Fos, and the first intron sequence of c-Fos mRNA (for nuclear c-Fos (nFos) staining) were isolated from mouse brain cDNA or mouse genomic DNA using PCR, and cloned into the pCR4 Topo vector (Thermo Fisher). Digoxigenin (DIG)- or dinitrophenol (DNP)-labelled cRNA probes (riboprobes) were prepared using the DIG RNA Labelling Mix (Roche) or DNP RNA labeling mix containing DNP-11-UTP (NEL555001EA, Perkin Elmer) and NTPs (Roche). Adult male C57BL/6J mice were perfused transcardially with 4% paraformaldehyde (PFA). Their brains were then soaked in 4% PFA for 4 h, in 30% sucrose for 48 h, and then frozen in OCT (Sakura) and cut into 20 μm coronal sections using a cryostat. Brains of CRH-Cre mice infected 3 days earlier with PRVB177, or wildtype animals, were fresh frozen in OCT, and cut into 20-μm coronal sections using a cryostat. Brain sections were hybridized to DIG- and/or DNP-labelled cRNA probes at 56 °C for 13–16 h.

Costaining for HA (PRVB177) and nFos mRNA. On d3pi of CRHNs with PRVB177, brain sections were hybridized with riboprobes for c-Fos and nFos as described previously^13^. After hybridization, sections were washed twice in 0.2 × SSC at 63 °C for 30 min, incubated with POD-conjugated anti-DIG antibodies (Roche, #11207733910, 1:2000) and biotinylated anti-HA antibodies (BioLegend, #901505, 1:300) at 37 °C for 2 h. Sections were then washed three times for 5 min at RT in TNT buffer, and then treated using the TSA-plus FLU kit (Perkin Elmer). Sections were then washed three times for 5 min at RT in TNT buffer and incubated with 0.5 μg/mL DAPI and Alexa555-Streptavidin (Thermo Fisher, #32355, 1:1,000) at room temperature for 1 h, and washed. Sections were coverslipped with Fluoromount-G (Southern Biotech).

Costaining for HA (PRVB177), nFos mRNA, and Vglut1/2 or Gad1/2 mRNA. On d3pi of CRHNs with PRVB177, sections were hybridized with DIG-labeled riboprobes for nFos and c-Fos and a DNP-labeled Vglut1/2 or Gad1/2 riboprobe. After hybridization, sections were washed twice in 0.2 × SSC at 63 °C for 30 min, incubated with POD-conjugated anti-DIG antibodies (Roche, #11207733910, 1:500), rabbit anti-DNP-KLH antibodies (Molecular Probes, #A6430, 1:500), goat anti-DNP antibodies (Bethyl Laboratories, #A150-117A, 1:500), and biotinylated anti-HA antibodies (BioLegend, #901505, 1:500) at 37 °C for 2 h. Sections were then washed three times for 5 min at RT in TNT buffer, and treated using the TSA-plus FLU kit (Perkin Elmer). Sections were then washed five times for 5 min at RT in TNT buffer and incubated with 0.5 μg/mL DAPI, Alexa555 Streptavidin (Thermo Fisher, #32355, 1:500), Alexa647 donkey anti-rabbit IgG (Thermo Fisher, #A21447, 1:500), Alexa647 donkey anti-goat IgG (Molecular Probes #A21447, 1:500) at 37 °C for 30 min, and washed. Sections were coverslipped with Fluoromount-G.

RNAscope was conducted using the RNAscope Multiplex Fluorescent Detection Kit v2 (ACDbio) following the manufacturer’s instructions with minor modifications. Briefly, sections were treated with RNAscope Protease Reagents for 30 min. After two washes in 1X PBS, sections were hybridized to Crh and c-Fos probes at 40 °C overnight. Bound probes were next amplified by RNAscope Multiplex FL v2 AMP at 40 °C. Sections were then incubated for 15 min at 40 °C with RNAscope Multiplex FL v2 HRPs followed by 30 min of incubation with Opal dyes. Sections were next incubated with DAPI and then coverslipped with Prolong Gold Antifade Mountant (Life Technologies).

#### Chemogenetic activation and silencing

Chemogenetic experiments were performed as described previously^13^ with some modifications.

#### Activation

AAV-DIO-hM3Dq-mCherry was injected into the VMH of Gad2-Cre mice by stereotaxic injection (see above). At 2 weeks after injection, mice were intraperitoneally injected with clozapine-N-oxide (CNO; Sigma) (5.0 mg/kg body weight) in 0.4% DMSO in saline or saline (0.4% DMSO dissolved in saline). Thirty minutes later, mice were exposed to restraint (see above). Trunk blood and brain were collected and the blood was used for plasma ACTH assays (see above). Brains were fixed by soaking in 4% PFA in PBS for 4 h, soaked in 30% sucrose for 24 h, frozen in OCT, and cut into 20-μm coronal sections using a cryostat. Brain sections were washed twice with PBS, permeabilized with 0.5% Triton X-100 in PBS for 5 min, washed twice with PBS, blocked with TNB (Perkin Elmer) for 1 h at room temperature, and then incubated with rabbit anti-RFP (to detect mCherry) (Rockland, #600-410-379, 1:500) diluted in TNB at 4 °C overnight. Sections were then washed three times with TNT, incubated with Alexa555 donkey anti-rabbit IgG (Thermo Fisher, #A31572, 1:1,000) and 0.5 μg/mL DAPI for 1 h at room temperature, and washed three times with TNT. Slides were coverslipped with Fluoromount-G (Southern Biotech).

#### Silencing

AAV-DIO-hM4Di-mCherry was injected into the VMH of Gad2-Cre mice by stereotaxic injection (see above). At 2 weeks after injection, mice were intraperitoneally injected with CNO (5.0 mg/kg body weight) or saline. Thirty minutes later, mice were exposed to restraint combined with odor exposure to 2PE (see above). Trunk blood and brain were then collected. Blood was used for plasma ACTH assays and brains were treated and immunostained with rabbit-anti-RFP antibody (see above).

#### Cell counting

Cell counting was performed as described previously^13^. Images were collected using an AxioCam camera and AxioImager.Z2 microscope with an apotome device (Zeiss). Images were acquired with auto-exposure setting, because backgrounds between different sections and animals can differ. No additional post-processing was performed on any of the collected images for counting. We used the stereological method of counting only cells that have a ‘unique identifier’, cells with nuclei stained by the nuclear dye, DAPI^28^. We counted colabeled cells based on the colocalization of signals for PRV and Vglut1/2 or Gad1/2 signals with only one nucleus. Therefore, we did not count two different cells as colabeled. Counting was conducted blindly. Brain structures were identified microscopically and in digital photos using a mouse brain atlas^29^. Every fifth section was analyzed for all experiments. For the data shown in Figs. 2 and 4, cells were counted in a given area in only one hemisphere, the hemisphere that contained the most PRV+ cells. The percentage of PRV+ cells with nFos labeling among all PRV+ cells lacking cytoplasmic Fos labeling was calculated.

### Statistical analysis

All data are shown as the mean ± S.E.M.. Data were tested with the Shapiro–Wilk test for normality. For data with a normal distribution, the unpaired t-test was used to compare two groups to analyze statistical significance. All tests were two-sided.

### Ethical approval

All methods were carried out in accordance with relevant guidelines and regulations.

## Supplementary Information

Supplementary Figures.

## Data Availability

All data needed to evaluate the conclusions in the paper are present in the paper. Additional data and materials related to this paper will be provided upon reasonable request (lbuck@fredhutch.org).

## Abbreviations

AH: Anterior hypothalamic area
ARC: Arcuate hypothalamic nucleus
AVPe: Anteroventral periventricular nucleus
BNSTa: Bed nucleus of the stria terminalis, anterior part
BNSTp: Bed nucleus of the stria terminalis, posterior part
DMH: Dorsomedial hypothalamic nucleus
LH: Lateral hypothalamic area
LPAG: Lateral periaqueductal grey
LPGi: Lateral paragigantocellular nucleus
LPO: Lateral preoptic area
LS: Lateral septal nucleus
MnPO: Median preoptic nucleus
MPA: Medial preoptic area
MPO: Medial preoptic nucleus
MTu: Medial tuberal nucleus
NTS: Nucleus of the solitary tract
PBN: Parabrachial nucleus
Pe: Periventricular nucleus of the hypothalamus
PH: Posterior hypothalamic nucleus
PLH: Peduncular part of lateral hypothalamus
PMV: Premammillary nucleus, ventral part
SCh: Suprachiasmatic nucleus
SHy: Septohippocampal nucleus
StHy: Striohypothalamic nucleus
VMH: Ventromedial hypothalamic nucleus
ZI: Zona incerta

## References

[1] Kandel E, Schwartz J, Jessell T, Siegelbaum S, Hudspeth AJ (2012). Principles of Neuroscience.

[2] Munger SD, Leinders-Zufall T, Zufall F (2009). Subsystem organization of the mammalian sense of smell. Annu. Rev. Physiol..

[3] Stowers L, Kuo TH (2015). Mammalian pheromones: Emerging properties and mechanisms of detection. Curr. Opin. Neurobiol..

[4] Lizarraga-Valderrama LR (2020). Effects of essential oils on central nervous system: Focus on mental health. Phytother. Res..

[5] Makara GB, Stark E, Karteszi M, Palkovits M, Rappay G (1981). Effects of paraventricular lesions on stimulated ACTH release and CRF in stalk-median eminence of the rat. Am. J. Physiol..

[6] Muglia L, Jacobson L, Majzoub JA (1996). Production of corticotropin-releasing hormone-deficient mice by targeted mutation in embryonic stem cells. Ann. N. Y. Acad. Sci..

[7] Pacak K, Palkovits M (2001). Stressor specificity of central neuroendocrine responses: Implications for stress-related disorders. Endocr. Rev..

[8] Schuurman T (1980). Hormonal correlates of agonistic behavior in adult male rats. Prog. Brain Res..

[9] Koike K (2021). Danger perception and stress response through an olfactory sensor for the bacterial metabolite hydrogen sulfide. Neuron.

[10] Ulrich-Lai YM, Herman JP (2009). Neural regulation of endocrine and autonomic stress responses. Nat. Rev. Neurosci..

[11] Kondoh K (2016). A specific area of olfactory cortex involved in stress hormone responses to predator odours. Nature.

[12] Hanchate NK (2020). Connect-seq to superimpose molecular on anatomical neural circuit maps. Proc. Natl. Acad. Sci. U.S.A..

[13] Lee EJ (2020). A psychological stressor conveyed by appetite-linked neurons. Sci. Adv..

[14] Root CM, Denny CA, Hen R, Axel R (2014). The participation of cortical amygdala in innate, odour-driven behaviour. Nature.

[15] Saraiva LR (2016). Combinatorial effects of odorants on mouse behavior. Proc. Natl. Acad. Sci. U.S.A..

[16] Li Q (2013). Synchronous evolution of an odor biosynthesis pathway and behavioral response. Curr. Biol..

[17] Murakami T (2012). Stress-related activities induced by predator odor may become indistinguishable by hinokitiol odor. NeuroReport.

[18] Kobayakawa K (2007). Innate versus learned odour processing in the mouse olfactory bulb. Nature.

[19] Matsukawa M, Imada M, Murakami T, Aizawa S, Sato T (2011). Rose odor can innately counteract predator odor. Brain Res..

[20] Frasnelli J, Schuster B, Hummel T (2007). Interactions between olfaction and the trigeminal system: What can be learned from olfactory loss. Cereb. Cortex.

[21] Willis DN, Morris JB (2013). Modulation of sensory irritation responsiveness by adenosine and malodorants. Chem. Senses.

[22] Krashes MJ (2014). An excitatory paraventricular nucleus to AgRP neuron circuit that drives hunger. Nature.

[23] Taniguchi H (2011). A resource of Cre driver lines for genetic targeting of GABAergic neurons in cerebral cortex. Neuron.

[24] Armbruster BN, Li X, Pausch MH, Herlitze S, Roth BL (2007). Evolving the lock to fit the key to create a family of G protein-coupled receptors potently activated by an inert ligand. Proc. Natl. Acad. Sci. U.S.A..

[25] Card JP, Enquist LW (2014). Transneuronal circuit analysis with pseudorabies viruses. Curr. Protoc. Neurosci..

[26] Krashes MJ (2011). Rapid, reversible activation of AgRP neurons drives feeding behavior in mice. J. Clin. Investig..

[27] Lee EJ (2011). Impairment of fear memory consolidation in maternally stressed male mouse offspring: Evidence for nongenomic glucocorticoid action on the amygdala. J. Neurosci..

[28] Schmitz C, Hof PR (2005). Design-based stereology in neuroscience. Neuroscience.

[29] Franklin K, Paxinos G (2008). The Mouse Brain in Stereotaxic Coordinates.

