# Supplementary material for: Odor blocking of stress hormone responses
Source: Sci Rep. 2022 May 24;12:8773. doi: 10.1038/s41598-022-12663-x (PMC9130126; doi:10.1038/s41598-022-12663-x)
Supplement: Supplementary file 1 — Supplementary Figures. [file 41598_2022_12663_MOESM1_ESM.pdf]

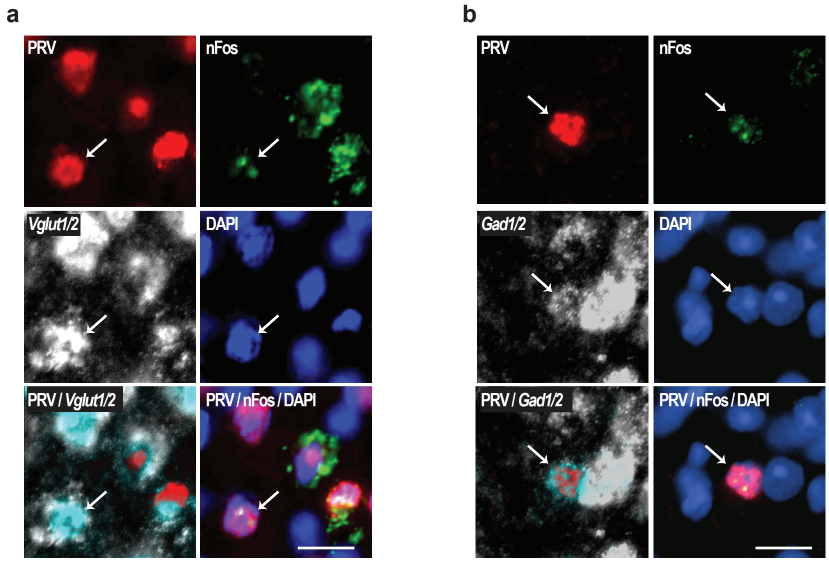

Supplementary Fig. 1

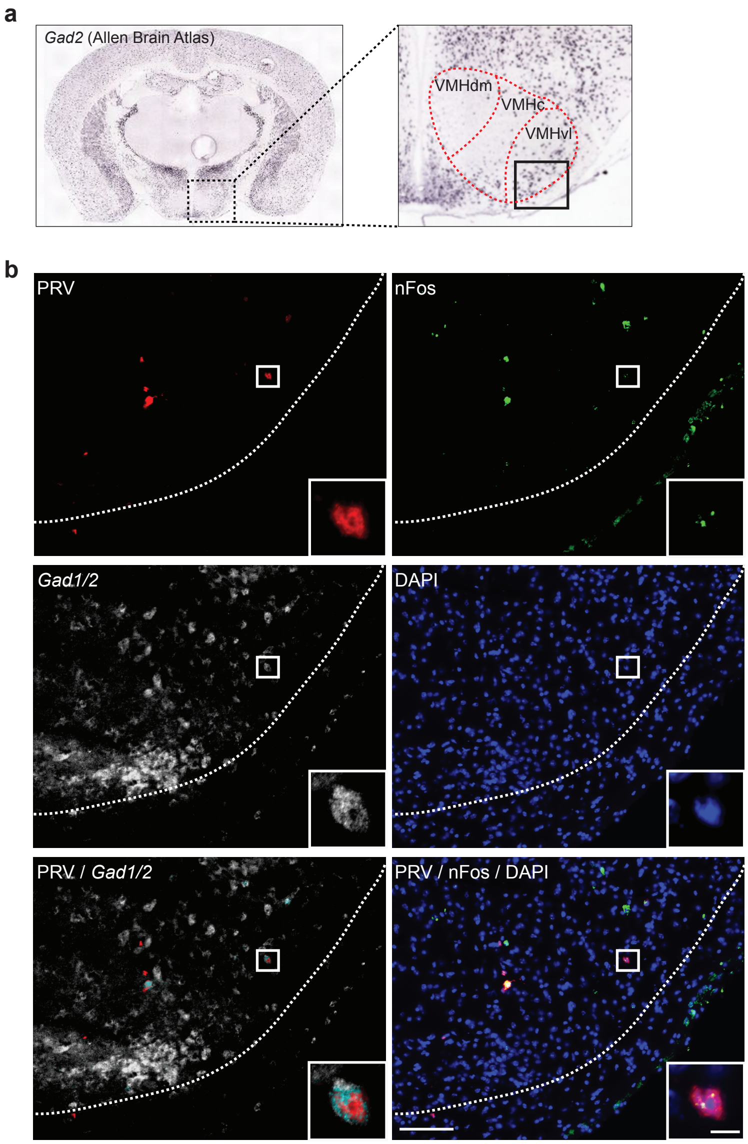

Supplementary Fig. 2

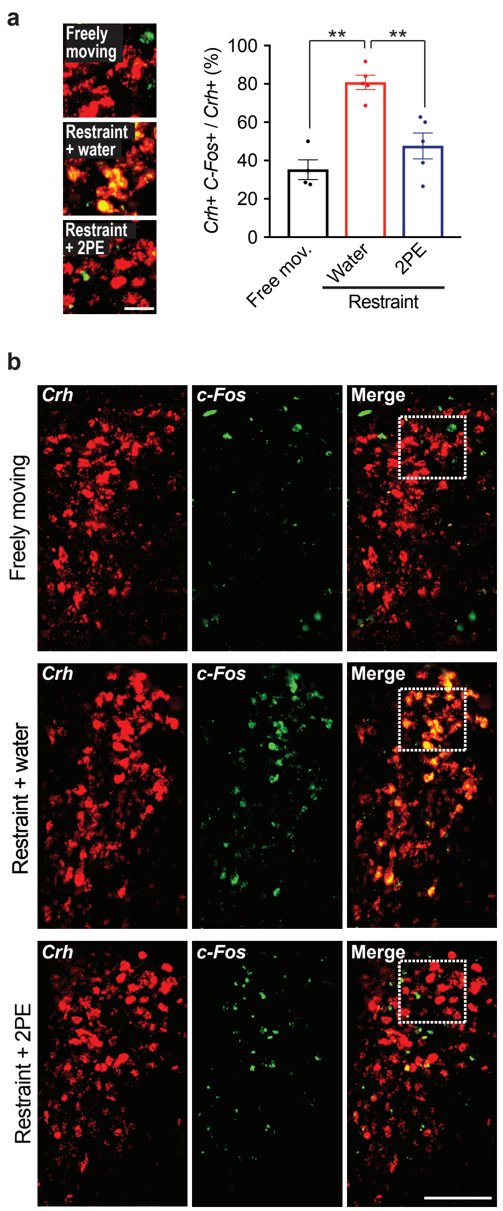

Supplementary Fig. 3

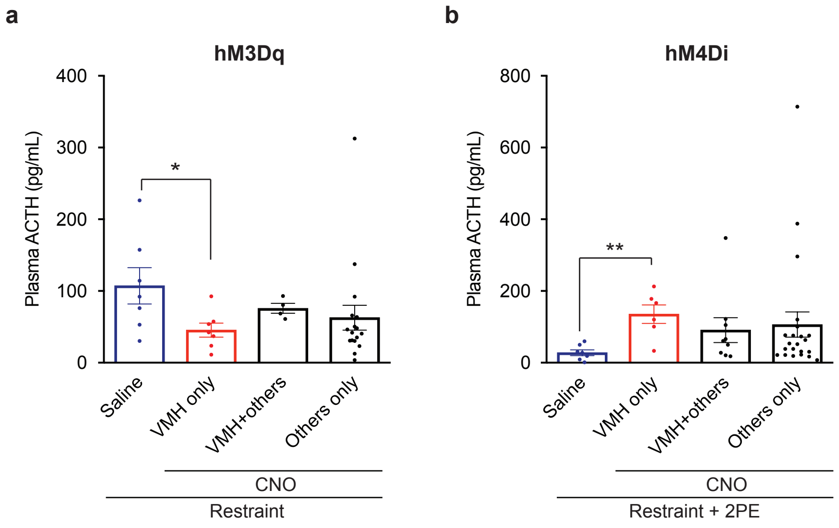

Supplementary Fig. 4

Supplementary Fig. 1. PRV+ neurons in BNSTa colabeled for nFos and *vGlut1/2* or *Gad1/2*

Following exposure to physical restraint, sections through the BNSTa were costained for PRV, nFos, and makers of glutamatergic neurons (*Vglut1/2*) (**a**) or GABAergic (*Gad1/2*) neurons (**b**) and then counterstained with DAPI. Photographic images show individual PRV+ neurons colabeled for nFos and *Vglut1/2* or *Gad1/2* (arrows). Scale bars, 20 µm.

Supplementary Fig. 2. Activation of upstream neurons in the VMH by 2PE

a. An image from the Allen Brain Atlas (https://mouse.brain-map.org/) shows staining for *Gad2* mRNA in the VMH. The dotted box at left is shown at higher magnification on the right with the VMH and its subregions outlined in red. The boxed area at right is shown in the photographic images below (**b**).

b. On day 3 after infection of CRHNs with PRVB177, animals were exposed to 2PE. Brain sections were then costained for PRV, and *Gad1/2* and nFos mRNAs, and counterstained with DAPI. Shown here are images of one VMH section showing labeling for PRV, nFos, *Gad1/2,* and DAPI. Merged images are also shown for PRV and *Gad1/2* and for PRV, nFos, and DAPI.

The boxed area is shown at higher magnification in the lower right corner of each image. The cell shown was labeled for PRV, nFos, and *Gad1/2*. Scale bars, 100 um (left), 10 um (right).

Supplementary Fig. 3. Activation of CRHNs by physical restraint

Brain sections were costained for *Crh* and *c-Fos* mRNAs in freely moving animals (Free mov.) or following exposure to physical restraint plus water or 2PE.

a. 2PE inhibited restraint-induced activation of CRHNs, as indicated by the percentage of *Crh*+ cells labeled for *c-Fos*. n=4-5 per condition. Unpaired t-test, **P<0.001.

b. Shown here are photographs of the paraventricular nucleus of the hypothalamus in each condition, with *Crh*+ neurons shown in red and *c-Fos*+ neurons in green. Merged images are shown at right. Boxes indicate segments shown at higher magnification at left in (a). Scale bars: 10 um in a, 100 um in b.

Supplementary Fig. 4. Activation or silencing of GABAergic neurons in VMH and/or nearby areas.

Cre-expressing Gad2+ neurons were infected with an AAV encoding a Cre-dependent activating receptor (hM3Dq) or silencing receptor (hM4Di) stimulated by CNO. Immunostaining for mCherry coexpressed with the receptors was used to locate cells infected with the AAVs. Some

animals showed infected neurons only in the VMH (VMH only), but others also had infected neurons in nearby areas (VMH+others) or only in nearby areas (Others only). Plasma ACTH was measured in animals treated with CNO or control saline and then exposed to physical restraint **(a)** or to physical restraint and 2PE **(b).**

a. In animals exposed to physical restraint, hM3Dq activation of Gad2+ neurons only in the VMH by CNO significantly inhibited restraint-induced increases in ACTH compared to saline treated controls. However, CNO treatment of animals expressing hM3Dq also or instead in nearby areas (DMH, ARC, LH, PLH, PH, MTu, or PMD) did not significantly inhibit restraint-induced increases in ACTH compared to saline treated controls. Column heights indicate means, error bars indicate S.E.M., and dots in the same column indicate different animals. n = 4–15 per condition. Unpaired *t* test, *P < 0.05.

b. In animals exposed to physical restraint plus 2PE, hM4Di silencing of Gad2+ neurons only in the VMH by CNO significantly increased plasma ACTH compared to saline treated controls. In contrast, hM4Di silencing of Gad2+ neurons in the VMH and also nearby areas or only in nearby areas by CNO did not show increases in plasma ACTH compared to saline treated controls. Column heights indicate means, error bars indicate S.E.M., and dots in the same column indicate different animals. n = 6–20 per condition. Unpaired *t* test, **P < 0.01.
